# Supplementary material for: Rapid turnover of life-cycle-related genes in the brown algae
Source: Genome Biol. 2019 Feb 14;20:35. doi: 10.1186/s13059-019-1630-6 (PMC6374913; doi:10.1186/s13059-019-1630-6)
Supplement: Supplementary file 3 — Protein alignments, corresponding Gblocks html file and CDS sequences for the lineage conserved gametophyte-biased genes. (ZIP 2109 kb) [file 13059_2019_1630_MOESM3_ESM.zip › Con_GA_alignments/Ec-27_001570.aa_ali.fasta-gb.htm]

Ec-27\_001570.aa.fasta.trans.aa\_ali.fasta


## Gblocks 0.91b Results

Processed file: **Ec-27\_001570.aa.fasta.trans.aa\_ali.fasta**  
Number of sequences: **2**  
Alignment assumed to be: **Protein**  
New number of positions: **233** (selected positions are underlined in blue)

```
                         10        20        30        40        50        60
                 =========+=========+=========+=========+=========+=========+
Ec-27_001570     MTGLLRRPHDFRLLRYASVSTKAHLLCGTSHQPLPQQVVEVNEDETSVCVRDAERPNKSI
MSTRG.21037.1    ------------------------------------------------------------
                                                                             


                         70        80        90       100       110       120
                 =========+=========+=========+=========+=========+=========+
Ec-27_001570     ECAFDKVFGCSSSQDDIFRGLVPALDTVVKGFNACVLAYGQTGSGKTHTLIGPGGGTRLA
MSTRG.21037.1    ------------------------------------------------------------
                                                                             


                        130       140       150       160       170       180
                 =========+=========+=========+=========+=========+=========+
Ec-27_001570     DDQTEWGIIPRAVQFLFDRLNLESEEDETFSYSVTCSFLQIYNENLVDLLLHSGGAAGAA
MSTRG.21037.1    ------------------------------------------------------------
                                                                             


                        190       200       210       220       230       240
                 =========+=========+=========+=========+=========+=========+
Ec-27_001570     PLQIREEAGGGDRGGRGGGGGHRNSVSSGGRRPWSNESSRGGEDETGGMNGEDVLFGAGY
MSTRG.21037.1    ------------------------------------------------------------
                                                                             


                        250       260       270       280       290       300
                 =========+=========+=========+=========+=========+=========+
Ec-27_001570     GKKNRASPVPKGGKANVFVNGLSSFRVGGAQDVLHYVNLGAANRRVRATQHNEASSRSHA
MSTRG.21037.1    ------------------------------------------------------------
                                                                             


                        310       320       330       340       350       360
                 =========+=========+=========+=========+=========+=========+
Ec-27_001570     VMQLTVEVKSGGRGGEEGGGGASDHITYRQAKLSLVDLAGSEKMDSALSISKGHFKELRS
MSTRG.21037.1    ------------------------------------------------------------
                                                                             


                        370       380       390       400       410       420
                 =========+=========+=========+=========+=========+=========+
Ec-27_001570     INQSLSTLGNVVSALSEKGRTHVPYRDSKLTRLLQDSLGGNTRTTIIACINPLAGQTHET
MSTRG.21037.1    ------------------------------------------------------------
                                                                             


                        430       440       450       460       470       480
                 =========+=========+=========+=========+=========+=========+
Ec-27_001570     INTLQFADRAKKVMVRLRANEVVDDRVLLARARAEIKRLKKRLREALEGTLLGTAAEDDN
MSTRG.21037.1    ------------------------------------------------------------
                                                                             


                        490       500       510       520       530       540
                 =========+=========+=========+=========+=========+=========+
Ec-27_001570     NDGHCPNESSRSCHNHDRNGTPEETKPCPETVDDVSPGGAGDGSARGVKEEGDKGHSMTT
MSTRG.21037.1    ------------------------------------------------------------
                                                                             


                        550       560       570       580       590       600
                 =========+=========+=========+=========+=========+=========+
Ec-27_001570     SAVSNNAAARRRSASPSKAAAAVPSTALRLPKFRKRQFAVDGGRGQDSGGEGEPVEDALT
MSTRG.21037.1    ------------------------------------------------------------
                                                                             


                        610       620       630       640       650       660
                 =========+=========+=========+=========+=========+=========+
Ec-27_001570     AEEVRVIVSNSAEAEGASGSPEQKEEEEEEEQDAEEAKQLAMFRREIQEGVVSGSLVKRG
MSTRG.21037.1    ------------------------------------------------------------
                                                                             


                        670       680       690       700       710       720
                 =========+=========+=========+=========+=========+=========+
Ec-27_001570     GGGGIGRGTATVPVKSGAAAGVDGDGDNDGDNVNGNPDEQADIEVFIDQSQRLEDLMFEA
MSTRG.21037.1    ------------------------------------------------------------
                                                                             


                        730       740       750       760       770       780
                 =========+=========+=========+=========+=========+=========+
Ec-27_001570     QGRERRRLRGTIEPRVGRFPPTPSPRTNANGGQPADAKMKVQQLQDQHEDRRGQQRRSPP
MSTRG.21037.1    ------------------------------------------------------------
                                                                             


                        790       800       810       820       830       840
                 =========+=========+=========+=========+=========+=========+
Ec-27_001570     CYDGALAAPDTCSLMATVAQPVLRALVSKEKGISINVGDDNGPVRTGWADQSSRTTRETH
MSTRG.21037.1    ------------------------------------------------------------
                                                                             


                        850       860       870       880       890       900
                 =========+=========+=========+=========+=========+=========+
Ec-27_001570     SITNVDPTPTPTPATTATTTAMMMAAVLASPETTTDGCEINDPHRRASSTNNSGINDNSA
MSTRG.21037.1    ----------------------------------------------------------XE
                                                                             


                        910       920       930       940       950       960
                 =========+=========+=========+=========+=========+=========+
Ec-27_001570     VEAFAGERVQRVVPAINPPLERRRRQRQTGTRNQSPIPSSPSSLHGHPFPSPSVQRRRPA
MSTRG.21037.1    LESLSGARAGRTVPATKPPLQRNRHGR-TERTDSSPTPPSPSSLSRRDSHLATSLRQRPA
                  ########################## ################           #####


                        970       980       990      1000      1010      1020
                 =========+=========+=========+=========+=========+=========+
Ec-27_001570     TSTGGPPTGRNRARSNSRRIYRR---KVGGSRSPVRGKPSAFLEPASPQVGPH-------
MSTRG.21037.1    TSTGYPPTGTSRAKSRSRRIRQRAGVRMGRSRSPIRAGREAFAEPASPQKRRIDGQRGGS
                 #######################     #####################           


                       1030      1040      1050      1060      1070      1080
                 =========+=========+=========+=========+=========+=========+
Ec-27_001570     ---DGGGEEHGPRRRQQGKVGS--------GGGGSGGGSSATKVVAATHPRLARSSPEQK
MSTRG.21037.1    QQHRAGQKQHQQRRQQQGNTGDKTLPARLMAAASSNENATIELQQAAGNPRLSSAPSEID
                      ################                        ###############


                       1090      1100      1110      1120      1130      1140
                 =========+=========+=========+=========+=========+=========+
Ec-27_001570     QRSGQEFLGADAD-------DQRKKGLSYSVADLGLRLKVYSFRYDHYYECQVVGYDSRR
MSTRG.21037.1    QKLGRKVGGSEASGAGAIEEQTTNRILNYSVADLGLRLKVYSFRYDHYYECQVVGYDSRR
                 ############              ##################################


                       1150      1160      1170      1180      1190      1200
                 =========+=========+=========+=========+=========+=========+
Ec-27_001570     RMHRCAYDATGEKQWHDLASKRFEVVGQNGVIVRSTESAPPAPA-----------SFATT
MSTRG.21037.1    RMHRCAYDATGERHWHDLANKRFEVSGQNGVKVRSTASAPPAPAPGRPPRDASKKSFSSR
                 ############################################                


                       1210      1220      1230      1240
                 =========+=========+=========+=========+====
Ec-27_001570     ATN--ATPSLPLPTSPSNIVGAITRESGGGHGAPGRKDMIKMYQ
MSTRG.21037.1    GSDESSNTPLLLPTSPRYPLGISFGNSLGGS-DVGSGGKL---R
                          #####################
```

```
Parameters used
Minimum Number Of Sequences For A Conserved Position: 2
Minimum Number Of Sequences For A Flanking Position: 2
Maximum Number Of Contiguous Nonconserved Positions: 8
Minimum Length Of A Block: 10
Allowed Gap Positions: None
Use Similarity Matrices: Yes
```

```
Flank positions of the 8 selected block(s)
Flanks: [902  927]  [929  944]  [956  983]  [989  1009]  [1026  1041]  [1066  1092]  [1107  1184]  [1210  1230]  

New number of positions in Ec-27_001570.aa.fasta.trans.aa_ali.fasta-gb:  233  (18% of the original 1244 positions)
```
